# Investigating heterogeneity across autism, ADHD, and typical development using measures of cortical thickness, surface area, cortical/subcortical volume, and structural covariance

**DOI:** 10.3389/frcha.2023.1171337

**Published:** 2023-09-27

**Authors:** Younes Sadat-Nejad, Marlee M. Vandewouw, R. Cardy, J. Lerch, M. J. Taylor, A. Iaboni, C. Hammill, B. Syed, J. A. Brian, E. Kelley, M. Ayub, J. Crosbie, R. Schachar, S. Georgiades, R. Nicolson, E. Anagnostou, A. Kushki

**Affiliations:** ^1^ Autism Research Centre, Bloorview Research Institute, Holland Bloorview Kids Rehabilitation Hospital, Toronto, ON, Canada; ^2^ Institute of Biomedical Engineering, University of Toronto, Toronto, ON, Canada; ^3^ Mouse Imaging Centre, The Hospital for Sick Children, Toronto, ON, Canada; ^4^ Program in Neuroscience and Mental Health, Department of Medical Biophysics, The Hospital for Sick Children, University of Toronto, Toronto, ON, Canada; ^5^ Nuffield Department of Clinical Neurosciences, Wellcome Centre for Integrative Neuroimaging, FMRIB, University of Oxford, Oxford, United Kingdom; ^6^ Diagnostic Imaging, The Hospital for Sick Children, Toronto, ON, Canada; ^7^ Department of Medical Imaging, University of Toronto, Toronto, ON, Canada; ^8^ Department of Paediatrics, University of Toronto, Toronto, ON, Canada; ^9^ Department of Psychology, Queen's University, Kingston, ON, Canada; ^10^ Centre for Neuroscience Studies, Queen's University, Kingston, ON, Canada; ^11^ Department of Psychiatry, Queen's University, Kingston, ON, Canada; ^12^ Department of Psychiatry, University of Toronto, Toronto, ON, Canada; ^13^ Department of Psychiatry, The Hospital for Sick Children, Toronto, ON, Canada; ^14^ Department of Psychiatry and Behavioural Neurosciences, McMaster University, Hamilton, ON, Canada; ^15^ Department of Psychiatry, Western University, London, ON, Canada

**Keywords:** neurodevelopmental conditions, autism, ADHD, clustering, brain structure, data driven

## Abstract

**Introduction:**

Attention-deficit/hyperactivity disorder (ADHD) and autism are multi-faceted neurodevelopmental conditions with limited biological markers. The clinical diagnoses of autism and ADHD are based on behavioural assessments and may not predict long-term outcomes or response to interventions and supports. To address this gap, data-driven methods can be used to discover groups of individuals with shared biological patterns.

**Methods:**

In this study, we investigated measures derived from cortical/subcortical volume, surface area, cortical thickness, and structural covariance investigated of 565 participants with diagnoses of autism [*n* = 262, median(IQR) age = 12.2(5.9), 22% female], and ADHD [*n* = 171, median(IQR) age = 11.1(4.0), 21% female] as well neurotypical children [*n* = 132, median(IQR) age = 12.1(6.7), 43% female]. We integrated cortical thickness, surface area, and cortical/subcortical volume, with a measure of single-participant structural covariance using a graph neural network approach.

**Results:**

Our findings suggest two large clusters, which differed in measures of adaptive functioning (*χ*^2^ = 7.8, *P* = 0.004), inattention (*χ*^2^ = 11.169, *P* < 0.001), hyperactivity (*χ*^2^ = 18.44, *P* < 0.001), IQ (*χ*^2^ = 9.24, *P* = 0.002), age (*χ*^2^ = 70.87, *P* < 0.001), and sex (*χ*^2^ = 105.6, *P* < 0.001).

**Discussion:**

These clusters did not align with existing diagnostic labels, suggesting that brain structure is more likely to be associated with differences in adaptive functioning, IQ, and ADHD features.

## Introduction

Autism spectrum disorder (hereafter autism) and attention-deficit/hyperactivity disorder (ADHD) are multifaceted neurodevelopmental conditions (1). Autism is characterized by differences in social communication and the presence of intense interests and repetitive behaviors (prevalence 1%–2%) (1); ADHD is defined by inattention and/or hyperactivity and impulsiveness (2) (prevalence 7%) (3). Currently, the diagnoses of autism and ADHD are based on observed and reported behavioral assessments (4), and no biological markers exist to inform the assignment of diagnostic labels. Despite this, the discrete labels of autism and ADHD are commonly used to inform service provision. However, these labels may not always capture the needs of neurodivergent populations (5). Within autism and ADHD, there is large variability in aetiology, neurobiology, phenotypic presentation, and profiles of strengths and disability (6, 7). At the same time, significant overlap exists between the two conditions. For example, the prevalence of co-occurring ADHD in autism is estimated to be 28% [95% CI: (25–32)] (8), while 21% [95% CI: (18–24)] of children with ADHD are reported to meet clinical thresholds for autism (9). Autism and ADHD are also reported to share genetic underpinnings (10) and neurobiological features including similarities in brain structure (11–13), function (14, 15), and connectivity (16, 17). Shared phenotypic and neurocognitive features also exist between autism and ADHD. These include differences in social communication (18), sensory processing (19), face processing (20), response inhibition and sustained attention (21). In this context, several studies have found that the labels of autism and ADHD may not be associated with unique and distinct biological and behavioural profiles (1, 12). The misalignment between these discrete labels and underlying neurobiology significantly challenges the development and provision of personalized supports that can appropriately fit the breadth of needs and strengths of neurodivergent individuals.

To address this gap, an emerging body of literature has focused on characterizing the heterogeneity in neurobiology and/or phenotypic presentation within and across neurodevelopmental conditions including autism and ADHD (10, 11–13). This approach shifts away from discrete diagnosis categories to a data-driven approach that can more closely capture profiles aligned with real-world outcomes (24). An example of this approach is the Research Domain Criteria (RDoc) framework (25). Data-driven approaches, and specifically clustering, have also received attention as they do not require any *a priori* assumptions of group labels; instead of examining differences between pre-defined diagnostic groupings, data-driven approaches look to the data to describe variability and discover homogeneous groupings that can map onto profiles of needs and strengths. The common finding across these studies is the lack of alignment between existing diagnostic labels and neurobiology, although the discovered subgroups vary significantly across different studies depending on the sample characteristics and data modality used [see review by Astle, Holmes, Kievit, and Cathercole (5)].

In terms of studies focused on neurobiology, measures of brain structure (12, 22), function (26), and connectivity (27) have been most commonly employed. The present work focuses on brain structure in particular. Brain structure is suggested to have utility as an intermediate phenotype that links multiple genetic variants (12, 23, 28), and gray matter volume differences have been documented in ASD and ADHD (29–32) compared to neurotypical populations. A key limitation of these studies, however, is that regional measures of brain structure are used in isolation and the relation between brain regions is often ignored in the analyses. This is a critical shortcoming as autism and ADHD are associated with pervasive differences across brain networks (33, 34). To address this limitation, the present study extends the previous literature by integrating regional associations in the form of structural covariance (35) into the clustering. Structural covariance quantifies population-level correlation among measures of gray matter morphology (36). Structural covariance networks are highly heritable, demonstrate alterations in samples of children with ASD (37) and ADHD (38, 39), have associations with cognitive ability (40), and replicate patterns of interregional functional and structural connectivity and maturational coupling in autism (41). We hypothesize that (a) data-driven clusters derived from sMRI data will not align with traditional diagnostic labels, demonstrated by low values of normalized mutual information, adjusted rand score, homogeneity, and completeness, (b) the derived clusters will transcend diagnostic boundaries and contain participants from different diagnosis groups, (c) there will be widespread cluster differences in brain structure across cortical and subcortical regions, and (d) the data-driven clusters will be associated with phenotypic differences across levels of cognition, behaviour, and function.

## Materials and methods

### Participants

The study used data from the Province of Ontario Neurodevelopmental Network (POND; export date: August 7, 2021). The dataset included participants with primary diagnoses of autism (*n* = 262) or ADHD (*n* = 171), as well as those who were neurotypical (TD) (*n* = 132) between the ages of 5–23 years (mean age: 12.2 ± 3.78; 413 males, 152 females). All participants and their parents were able to complete the testing protocols in English and had no contraindications to magnetic resonance imaging (MRI). Participants in the clinical groups met the DSM criteria for their respective diagnosis and diagnoses were supported by gold-standard assessments [autism: Autism Diagnostic Observation Schedule–2 (ADOS) (42) and Autism Diagnostic Interview–Revised (ADI-R) (20); ADHD: Parent Interview for Child Symptoms (PICS) (43)]. Participants in the TD group did not have a neurodevelopmental, psychiatric and/or neurological diagnosis or any first-degree relatives with a neurodevelopmental condition, and were born after 35 weeks gestation. Informed consent was provided by participants (when they had the capacity to consent) or their guardians, and assent was obtained from all participants as per institutional ethics board guidelines. Institutional research ethics boards approvals were received for the study.

### Behavioral measures

Participants were characterized using phenotypic measures quantifying core and co-occurring conditions. These included the Social Communication Questionnaire (SCQ; lifetime) (44) for autism-like traits (total score) and the Strengths and Weaknesses of ADHD-symptoms and Normal Behavior (SWAN) (45) for ADHD-like features (inattentive and hyperactivity subscales). Adaptive functioning was quantified using the Adaptive Behavior Assessment System-II (ABAS-II) (46) for adaptive function (general ability composite). Co-occurring emotional and behavioural symptoms were quantified using the Child Behavior Checklist (CBCL) (47). Neurophysiological assessments of participants included the Developmental Neuropsychological Assessment (NEPSY; affect recognition and memory for faces) (48) as well as the stop-signal task (response inhibition, sustained attention, and reaction time) (21). Full-scale IQ was measured using the Wechsler Abbreviated Scale of Intelligence (WASI) (49), the Wechsler Intelligence Scale for Children (WISC) (50), or the Stanford-Binet Test (51) as appropriate for age and ability level.

### Imaging data

We used structural MRI (sMRI) to extract measures of surface area, cortical thickness, and cortical/subcortical regions volume. A portion of images were collected on a 3-Tesla Siemens Trio TIM at the Hospital for Sick Children (*n* = 280), which was upgraded to a Siemens PrismaFIT scanner (*n* = 395). The rest of the data were collected using 3-Tesla Siemens Trio TIM at Queen's University (*n* = 113) which was later updated to Siemens PrismaFit scanner (*n* = 5). The parameters for Imaging acquisition are provided in Supplementary Table S1. The CIVET pipeline (version 2.1.0) (52) was used to extract area, cortical thickness, and cortical volume for 76 regions of interest (ROI) according to automated anatomical labeling atlas (AAL) (53, 54) using the T1-weighted images. The pipeline includes non-uniformity image correction followed by stereotaxic registration to the Montreal Neurologic Institute (MNI ICBM) (55) template (non-linear 6th generation target) (56). Furthermore, gray matter, white matter, and cerebrospinal fluid were obtained by the process of masking, extraction, and classification. Gray matter and white matter surfaces were then created using tissue classification images (57–60). A surface diffusion kernel (44), followed by registration of regions to the AAL (53, 54) was applied. To calculate cortical thickness, the distance between two smoothed surfaces (61) was used. Surface area was generated by tissue classification images (41). Subcortical structure volumes (95 regions) were calculated based on segmentation using multiple automatically generated templates (MAGeT) (62). A list of regions included in the analysis is provided in Supplementary Table S2. The CIVET and MAGeT quality control (QC) pipelines were used. The CIVET QC pipeline excludes scans with artifacts, inaccurate segmentations, and registration errors (52). Motion artifacts are detected based on the number of surface to surface intersections per hemisphere (limit of 150 touch points per surface pair per side). MAGeT's QC pipeline removes data with missing values (63). The data were corrected for scanner effects using the ComBat Harmonization model (53), for sex (64) using linear regression, and for age using the best fitting linear, quadratic, or cubic polynomial models determined through cross validation.

### Analysis

#### Pipeline

**Figure 1:**
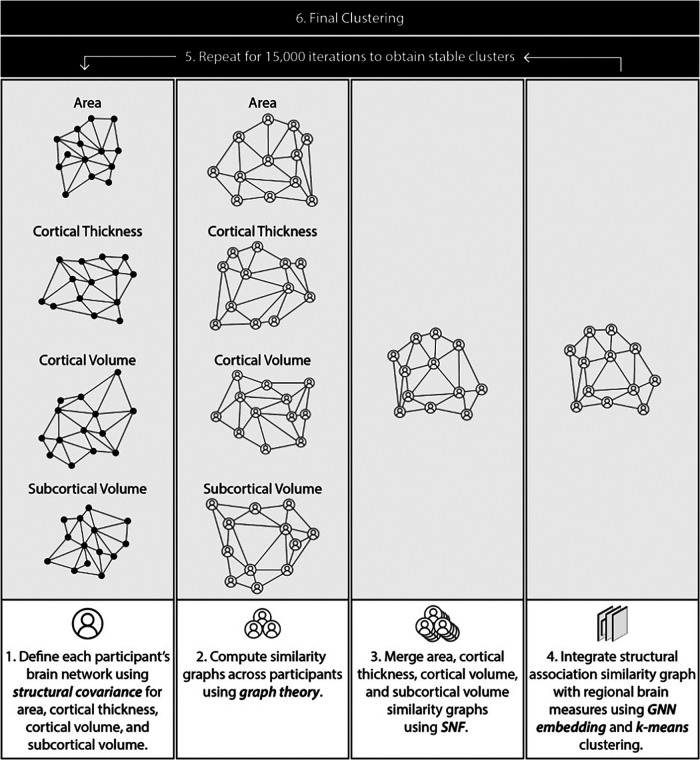
Overview of methodology pipeline.

Structural MRI data were processed using an analytical pipeline to discover clusters based on regional volume, cortical thickness, surface area, as well as associations between pairs of regions. To this end, we first computed statistical associations among regions for each participant (“participant-level association graph”). Next, the participant association graphs were used to compute similarity among pairs of participants resulting in a participant similarity matrix. This similarity matrix was then integrated with regional structural features to generate a final similarity matrix which was used for clustering. Preprocessing and clustering were carried out in Python 3.8.0. An overview of the pipeline is described below and summarized in Figure 1. A detailed description of the pipeline is provided in the Supplementary Materials.

##### Compute single participant structural association graphs (participant association graphs)

Structural covariance for a pair of regions is typically computed using the entire participant pool. We extended this concept, to compute a measure of statistical association between two regions for a single participant. To this end, we computed the linear regression line for a pair of regions using the entire participant pool (as done in structural covariance computation). For each participant, we then computed a measure of deviation from this line using the Cook's distance (65). The distance was transformed into a similarity value using a Guassian kernel. This process was repeated for all region pairs, resulting in a single graph for each participant with regions as nodes and the computed similarity as edge weights. Each brain measure was treated independently, leading to four association graphs per participant (surface area, cortical thickness, cortical volume, and subcortical volume).

##### Compute a participant similarity graph

Participant association graphs from step 1 were used to compute the similarity between pairs of participants. This was done using the Gaussian-transformed Lambda distance (66, 67). The participant similarities were represented as a graph with participants as nodes and similarities as edge weights.

##### Merge similarity matrices

Step 2 generated four participant similarity matrices, one for each brain measure (cortical thickness, surface area, cortical and subcortical volume). These were merged using Similarity Network Fusion (SNF) (68), resulting in a single integrated participant similarity graph.

##### Integrate structural association similarity with regional brain measures

The similarity graph in step 2 was built using statistical associations between regions. Next, we integrated this matrix with information about regional measures of cortical thickness, surface area, and cortical and subcortical volume. This was accomplished using a Graph Neural Network (GNN) as described in the Supplementary Materials. K-means clustering (69) was used to cluster the compact representation of each participant derived in step 4. To reduce sensitivity of the clustering solution to choice of parameters, this step was repeated with varying the number of clusters and SNF parameters (scaling factor µ, number of neighbours K), and consensus between resultant clusters across 15,000 iterations was used to derive final clusters (27) (detailed description of methods in Supplementary Methods). The Davies Bouldin score (70), Silhouette Coefficient (71), and Calinski-Harabasz score (72) were used to find the optimal number of clusters.

#### Post-hoc statistical analyses

We compared the result of our clustering with the clinical diagnostic labels using normalized mutual information (73), adjusted rand score (74), homogeneity, and completeness (75). All measures provide a score between zero (low) and one (high). Phenotypic and brain characteristics were compared across clusters using the *t*-test or Kruskal–Wallis test for normally and non-normally distributed continuous data, respectively. For categorical data, the chi-squared test was used. Statistical analyses were performed in R 3.3.3.

## Results

### Participants

From a total of 661 participants, 79 failed quality control (QC) for CIVET and of the remaining participants 17 participants failed QC for MAGeT (breakdown by diagnosis and age group is provided in Supplementary Tables S3, S4). The demographic characteristics for the remaining 565 participants are shown in Table 1. As seen, the autism and ADHD groups had a significantly higher proportion of males than females (*χ*^2^ = 25.40, *P* < 0.0001), and a significant age difference between ADHD, autism, and TD groups was observed *χ*^2^ = 7.28, *P* = 0.026). Table 1 also provides the clinical characterization of the participants. A subset of the participants also had neurocognitive assessments (NEPSY and stop-signal task) available. This information is reported in Supplementary Table S5. Medication usage in the sample is reported in Supplementary Table S6.

**Table 1:** Participant demographics.

	Autism (*n* = 262)	ADHD (*n* = 171)	TD (*n* = 132)	Group effect (corrected *P*-value)
Age	12.2 (5.99)	11.1 (4.00)	12.1 (6.75)	0.026 (TD > ASD > ADHD)
Sex (m: f)	204: 58	135: 36	74: 58	<0.0001
SCQ	20.0 (10.00)	5.0 (7.00)	2.0 (2.25)	<0.0001 (ASD > ADHD > TD)
SCQ ≥ 15 (%)	76.2%	12.2%	0%	<0.0001 (ASD > ADHD > TD)
Able to talk in short phrases or sentences (SCQ 1; %)	98.3%	100%	99.2%	0.074
Able to have a to and from conversation (SCQ 2; %)	74.81%	93.57%	92.42%	<0.0001 (ASD < ADHD, TD)
SWAN—inattention	5.0 (5.00)	6.0 (5.00)	0.0 (0.00)	<0.0001 (TD < ASD,ADHD)
% SWAN-inattention >6 (%)	42.8%	61.81%	0%	<0.0001 (ADHD > ASD > TD)
SWAN—hyperactivity	3.0 (5.00)	3.0 (6.00)	0.0 (0.00)	<0.0001 (TD < ASD,ADHD)
% SWAN-hyperactivity >6 (%)	27.51%	35.15%	0%	<0.0001 (ADHD > ASD > 0)
ABAS-II	65.0 (20.00)	80.0 (23.00)	105.0 (19.00)	<0.0001 (TD > ADHD > ASD)
CBCL internalizing	65 (12.75)	62 (15.5)	48 (14)	<0.0001 (ASD > ADHD > TD)
CBCL internalizing >65 (%)	54.8%	44.92%	8.51%	<0.0001 (ASD > ADHD > TD)
CBCL externalizing	58.5 (15.50)	61.0 (15.75)	43.0 (16.00)	<0.0001 (ADHD > ASD > TD)
CBCL externalizing >65 (%)	27.43%	40.96%	1.80%	<0.0001 (ADHD > ASD > TD)
Full-scale IQ	95.0 (27.25)	101.0 (19.50)	109.0 (17.25)	<0.0001 (TD > ADHD > ASD)
Household income (low: medium: high)	41: 72: 23	20: 31: 16	19: 49: 43	0.638
Household Education (no degree: high school: associate: undergraduate: graduate)	5: 7: 52: 79: 33	2: 3: 26: 39: 25	1: 6:3 7: 63: 50	<0.0001
Ethnicity: White (%)	50.3%	63.5%	74.1%	0.063
Ethnicity: South Asian (%)	1.1%	2.1%	6.8%	0.235
Ethnicity: Middle Eastern (%)	1.8%	1.0%	1.9%	0.740
Ethnicity: Latino (%)	3.5%	5.7%	6.2%	0.929
Ethnicity: Indigenous (%)	7.0%	4.7%	0.6%	0.005
Ethnicity: South East Asian (%)	0.7%	0%	3.1%	0.049
Ethnicity: East Asian (%)	4.2%	2.6%	8.0%	0.239
Ethnicity: Black (%)	3.8%	2.6%	5.6%	0.322

Reported values are median [interquartile range (IQR)]. ABAS, adaptive behavior assessment system-II; CBCL, child behaviour checklist; SCQ, social communication questionnaire; SWAN, strengths and weaknesses of ADHD-symptoms and normal behavior.

### Clusters

**Figure 2:**
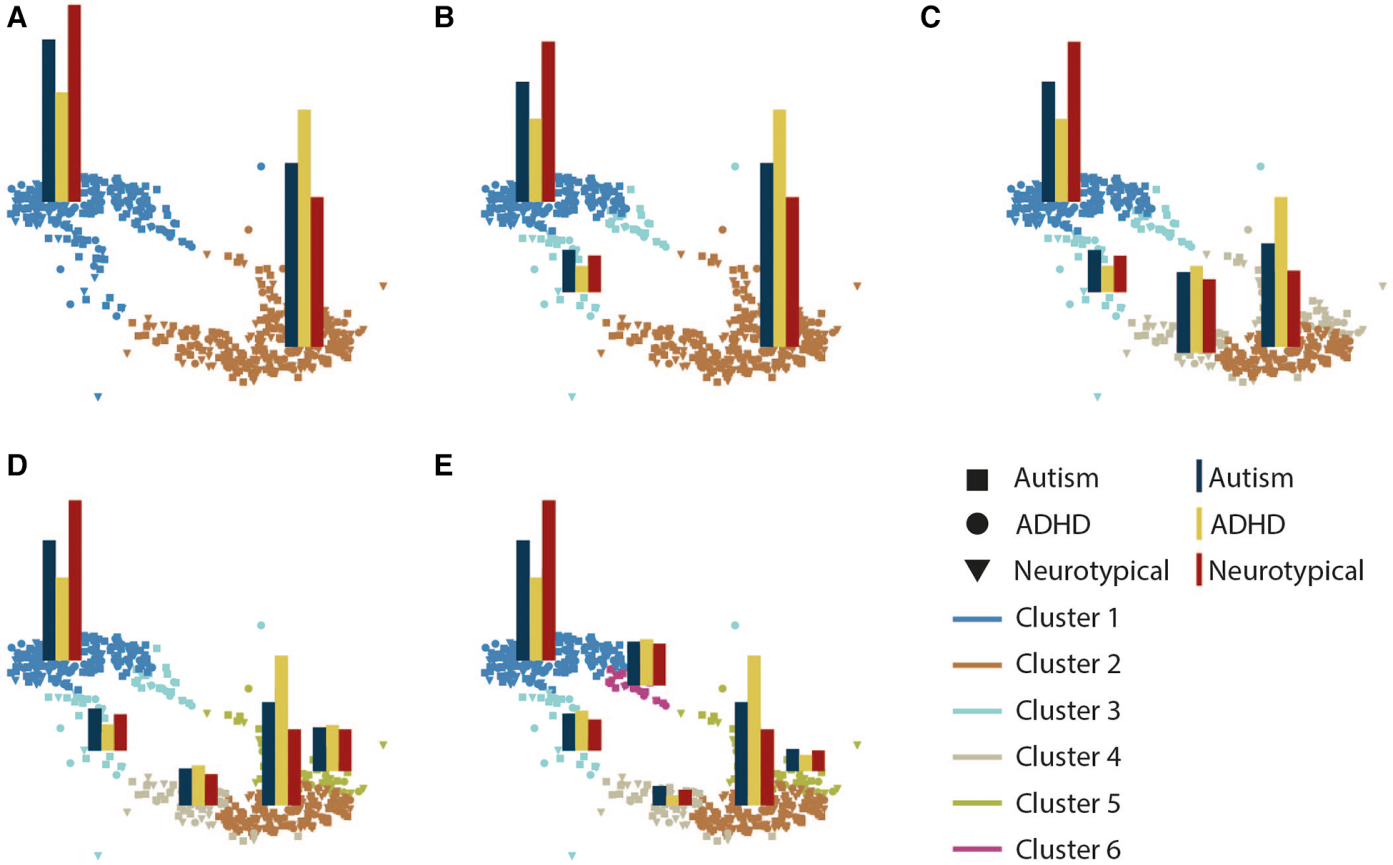
Graph representation of clusters as the number of clusters increases from 2 (**A**) to 6 (**E**). Each subplot illustrates a graph representation with participants as nodes. Edges are not displayed to enhance readability. Node colors distinct clusters, and superimposed bar plots show the distribution of diagnostic groups within each cluster (autism, ADHD, TD).

The Davies Bouldin score (70), Silhouette Coefficient (71), and Calinski-Harabasz score (72) all identified the 2-cluster solution as optimal; However, to enhance the generalizability of the results, we present the clustering solution for 2 to 6 clusters (Figure 2). As seen, participants were grouped into two clusters (cluster 1: *n* = 252, cluster 2: *n* = 313), although both groups had mixed representation from the diagnostic groups (TD: autism: ADHD; cluster 1: 57%, 47%, 32%; cluster 2: 43%, 53%, 68%), cluster 1 contained the majority of the TD participants (57%) whereas cluster 2 contained the majority of participants with a diagnosis of ADHD (68%). We, therefore will refer to these clusters as TD- and ADHD-enriched clusters, respectively. As we increased the number of clusters, cluster structure remained stable with the tails of the two clusters separating into new clusters (Figure 2). To examine the alignment between diagnostic labels and our cluster assignments (Supplementary Figure S1), we computed the normalized mutual information and adjusted rand scores. The scores were less than 0.02 and 0.01 consistently as the number of clusters varied from 2 to 6, indicating low agreement between the clusters and existing diagnostic labels. Furthermore, the homogeneity and completeness scores were both less than 0.02, demonstrating that obtained clusters did not represent a single diagnostic group regardless of the number of clusters used.

#### Brain correlates of clusters

**Figure 3:**
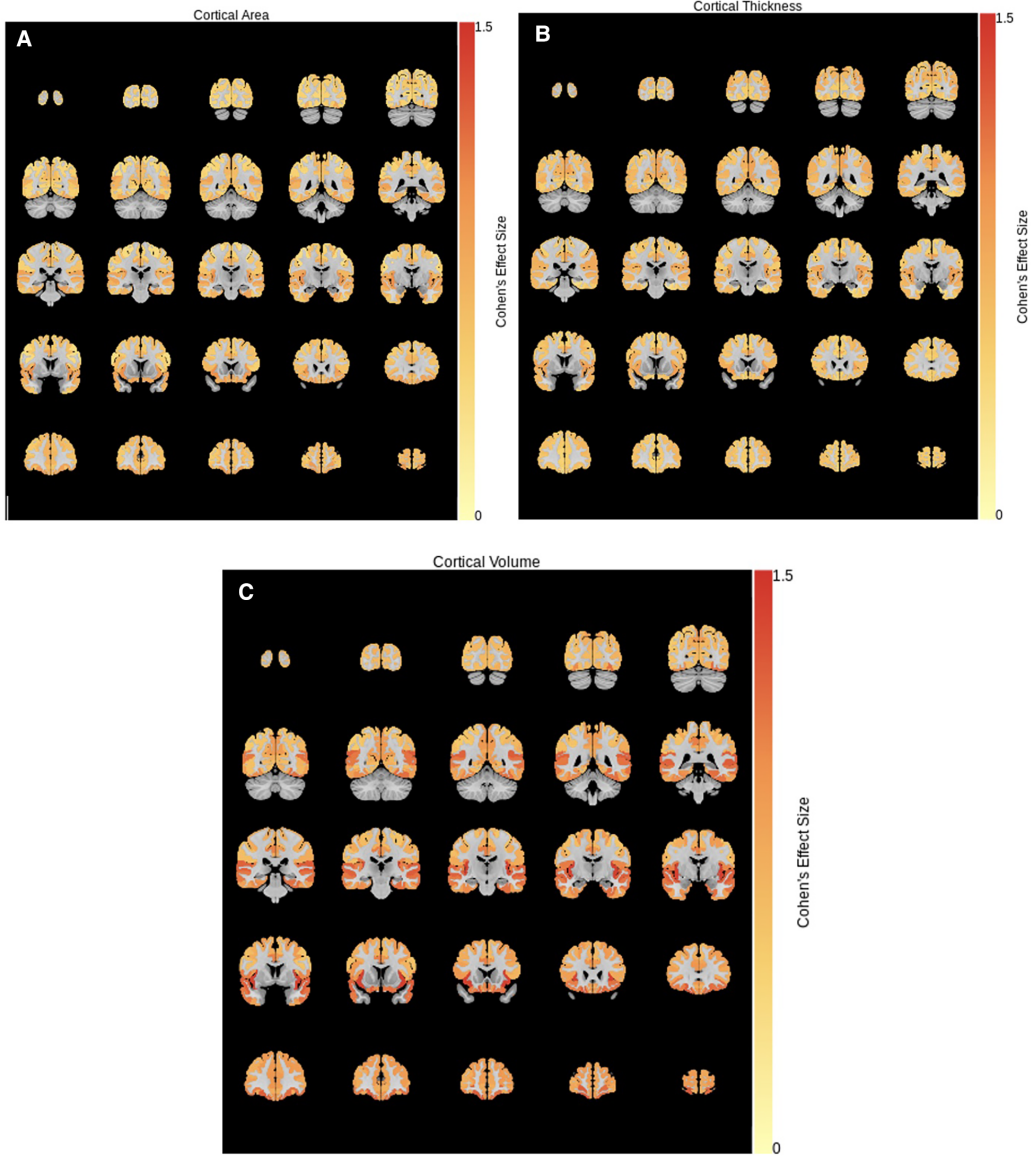
Cohen's effect size for cluster differences (TD-enriched—ADHD-enriched) in (**A**) surface area, (**B**) cortical thickness, and (**C**) cortical volume.

The two clusters were compared on measures of cortical and subcortical volume, cortical thickness, and surface area. Cohen's effect size is shown for regions where significant differences were found after correction for multiple comparisons in Figure 3. As seen, the largest effect sizes were found in cortical volume (TD-enriched > ADHD-enriched) across the cortex. Volumes of subcortical regions were not significantly different between the two clusters, except in the cerebellar vermis III and VIIB (ADHD-enriched > TD-enriched).

**Figure 4:**
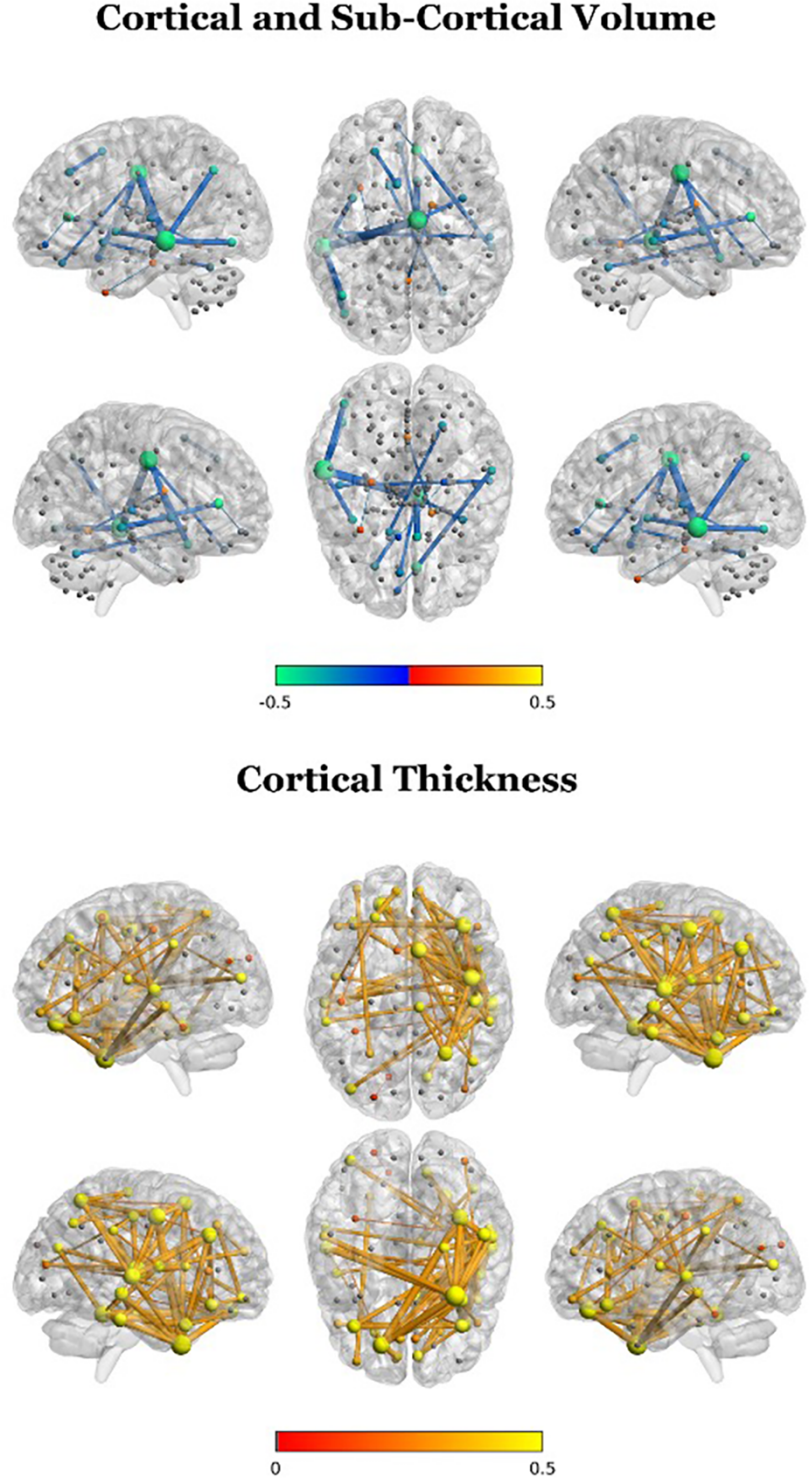
Cluster differences in edge weights for participant brain networks (2 clusters; TD-enriched—ADHD-enriched). A positive difference in this plot indicates that the statistical association between two given regions were more closely aligned with the overall sample structural covariance for the TD-enriched cluster compared to the ADHD-enriched cluster.

To further characterize cluster differences, we compared the two clusters in structural association edge weights using Kruskal–Wallis tests. After FDR correction for multiple comparisons, edge weight values were significantly different between the two clusters for cortical volume, cortical thickness, and subcortical volume for several regions, but not for surface area (Figure 4). For cortical volume, negative differences (TD-enriched < ADHD-enriched) were observed for several regions pairs, with the left middle temporal gyrus featuring most prominently (associations with the the right median cingulate and paracingulate gyri, left angular gyrus, and left inferior occipital gyrus, left insula). Association between the right cingulate gyri and various regions were also found to be significant (right median cingulate and right olfactory cortex, right insula, left gyrus rectus; right anterior cingulate and right middle temporal gyrus). Negative differences (TD-enriched < ADHD-enriched) were also found in subcortical regions, including in the associations between the left nucleus accumbens/ventral striatum and right cerebellum VI, and the right postcommissural caudate and vermal IV, suggesting closer alignment of the ADHD-enriched cluster with the sample trend.

For cortical thickness, widespread positive differences (TD-enriched > ADHD-enriched) were found, mainly in the right hemisphere. Most prominently, these included associations between the right fusiform gyrus and the right temporal gyri and the left superior frontal gyrus as well as associations among frontal and temporal gyri.

#### Demographic/phenotypic characterization of clusters

**Figure 5:**
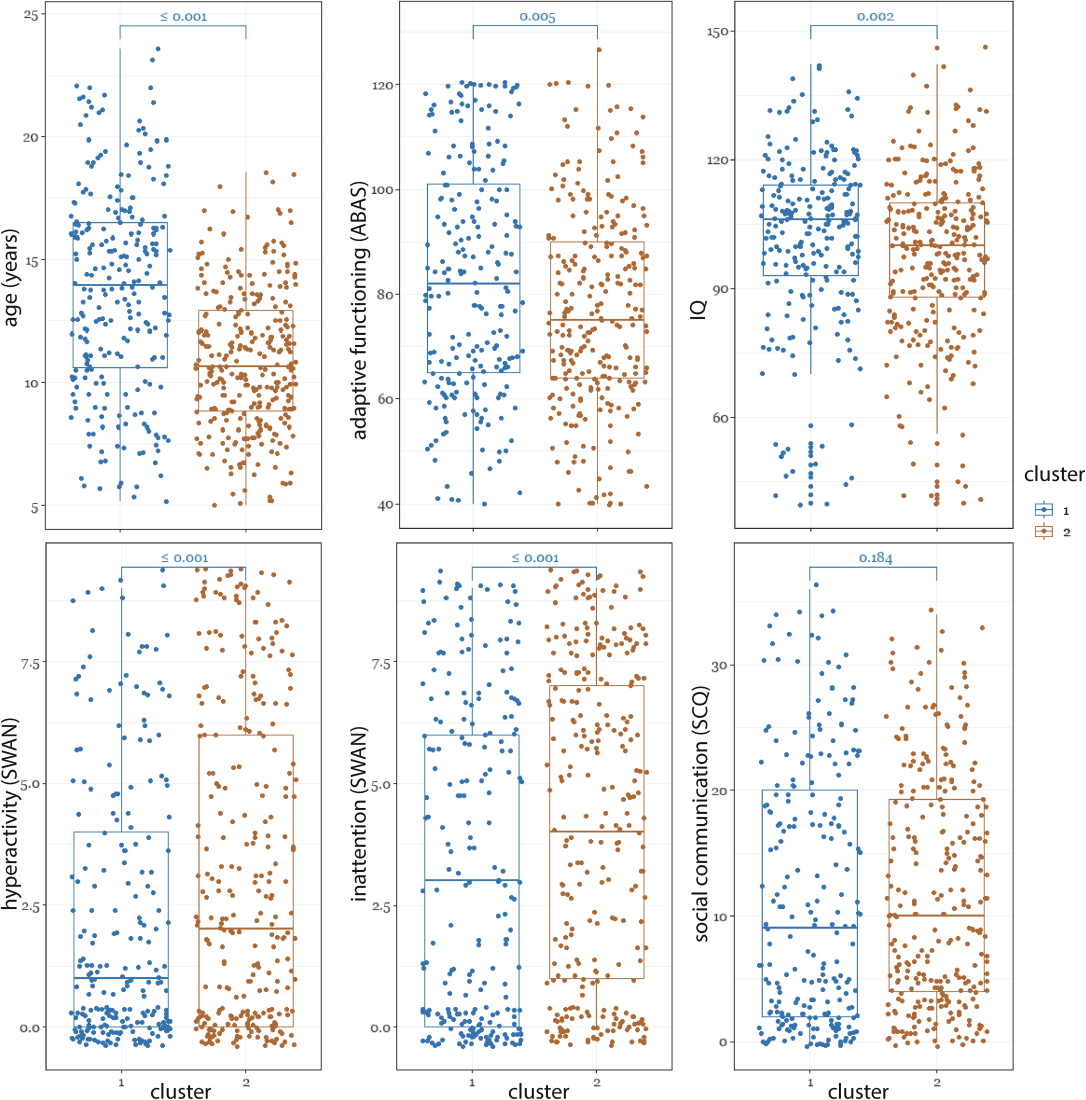
Comparison of phenotypic measures between the two clusters.

The demographic and phenotypic differences between the two clusters are shown in Figure 5. Compared to the TD-enriched cluster, the ADHD-enriched cluster had significantly higher proportion of ADHD participants (ADHD-enriched: 53.9% ADHD, TD-enriched: 46.1% ADHD; *χ*^2^ = 16.08, *P* < 0.0001) and lower proportion of TD (ADHD-enriched: 15.8% ADHD, TD-enriched: 84.2% ADHD; *χ*^2^ = 9.77, *P* = 0.0017). The participants in the ADHD-enriched cluster were significantly younger than those in the TD-enriched group (median difference = 3.3; *χ*^2^ = 70.84, *P* < 0.0001). As expected, this cluster also contained a significantly higher proportion of males (ADHD-enriched: 68.5%, TD-enriched: 31.5%; *χ*^2^ = 105.60, *P* < 0.0001) and had significantly higher median scores on ADHD-like features [SWAN inattention: ADHD-enriched: 4.0(6.00), TD-enriched: 3.0(6.00); *χ*^2^ = 11.20, *P* < 0.0001; SWAN hyperactivity -ADHD-enriched: 2.0(6.00), TD-enriched: 1.0(4.00); *χ*^2^ = 18.40, *P* < 0.0001], lower median scores on adaptive functioning [ABAS-General Ability Composite: ADHD-enriched: 75.0(26.00), TD-enriched: 82.0(36.00), *χ*^2^ = 7.80, *P* = 0.0052], lower inhibitory control [Stop Task stop reaction time: ADHD-enriched: 244.8(141.82), TD-enriched:292.17(130.31), *χ*^2^ = 7.80, *P* = 0.0009], and lower IQ [ADHD-enriched: 100.0(22.00), TD-enriched: 106.0(21.00), *χ*^2^ = 14.06, *P* = 0.0024]. NEPSY memory for faces and affect recognition, SCQ, CBCL internationalizing, and CBCL externalizing scores were not significantly different between clusters.

**Figure 6:**
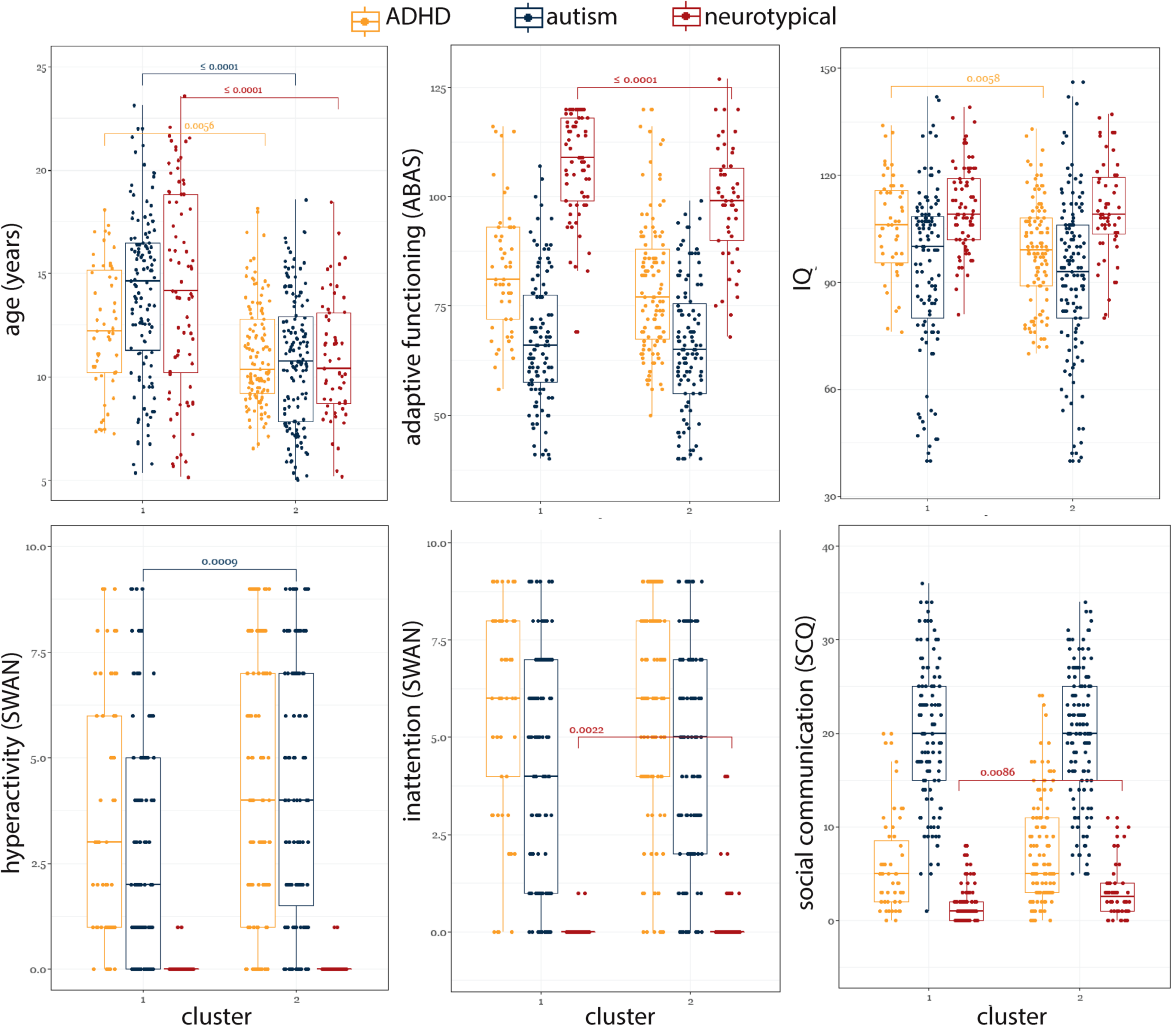
Comparison of participants with the same diagnosis across two clusters.

To gain further insight into the misalignment between diagnoses and our clusters, we investigated differences among participants who had the same diagnostic label (autism, ADHD, and TD) but fell into different clusters (Figure 6). Compared to those in the ADHD-enriched cluster, TD participants in the TD-enriched cluster were significantly older [ADHD-enriched: 14.1(8.63), TD-enriched: 10.4(4.34), *χ*^2^ = 15.75, *P* < 0.0001], had significantly lower SCQ scores [ADHD-enriched: 2.5(3.00), TD-enriched: 1.0(2.00); *χ*^2^ = 6.91, *P* = 0.0086], and higher ABAS scores [ADHD-enriched: 99.0(19.00), TD-enriched: 109.0(16.50); *χ*^2^ = 15.05, *P* = 0.0001]. Participants with ADHD in the ADHD-enriched cluster were also younger than those in the TD-enriched cluster [ADHD-enriched: 10.4(3.59), TD-enriched: 12.2(4.98); *χ*^2^ = 7.69, *P* = 0.006], and had lower IQ scores [ADHD-enriched: 99.0(19.00), Mixed: 106.0(20.25); *χ*^2^ = 7.61, *P* = 0.006]. Autistic participants in ADHD-enriched cluster were also significantly younger [ADHD-enriched: 10.7(5.07), TD-enriched: 14.6(5.19), *χ*^2^ = 45.46, *P* < 0.0001], and had a significantly higher SWAN-hyperactivity score [ADHD-enriched: 4.0(5.50), TD-enriched: 2.0(5.00); *χ*^2^ = 11.03, *P* = 0.0009].

## Discussion

Using a data-driven and diagnosis-agnostic approach, this paper characterized the variability in brain structure across neurodiverse children and youth. We introduced a novel analytics pipeline that integrated interregional couplings in addition to other measures of brain structure into the clustering algorithm. This is particularly critical as autism and ADHD are associated with differences in brain networks, and not specific regional lesions (13, 76–79).

Consistent with the previous literature (11, 13) examining measures of brain function and structure, our results revealed a mismatch between biological homogeneity and the labels of autism, ADHD, and typical development; instead, we found clusters that contained participants from different diagnostic groups. This finding lends further support to the idea that these discrete categories do not represent neurobiologically homogeneous groups. This motivates the need for an approach that characterizes neurodevelopmental diversity in a way that can identify dimensions of real-world needs (24).

Our results suggest the presence of two clusters which were neurobiologically different in brain structure. The first cluster was characterized by larger cortical volumes across the brain. This cluster was enriched for TD participants and, on average, was associated with decreased autism and ADHD traits, older age, and increased adaptive functioning and IQ. The second cluster was enriched for participants with a diagnosis of ADHD and associated with increased inattention and hyperactivity, and lower IQ and adaptive functioning. These results suggest that our clustering results are driven by features across multiple domains of cognition, behaviour, and function. In particular, our ADHD-enriched cluster was associated with decreased response inhibition [i.e., longer stop signal reaction time (SSRT)], increased ADHD symptoms (inattention and hyperactivity), and decreased adaptive functioning. Decreased response inhibition is a well-established neurocognitive feature of ADHD (80), but the findings in autism have been mixed (81). There is emerging evidence to suggest that response inhibition differences in autism may be explained by co-occurring ADHD symptoms (21), a finding that is consistent with our results. The clusters were not different in autism-like features at the cognitive (NEPSY affect recognition, memory for faces) or behavioural (SCQ) levels, suggesting specificity of the clustering to ADHD-like traits. These findings should be interpreted with caution, however, given that SSRT decreases with age and the fact that our ADHD-enriched cluster had lower average age (21). This is also supported by our finding of significant differences between participants who shared the same diagnosis label, but were assigned to different clusters. In particular, participants from the TD group who were assigned to the ADHD-cluster had higher scores on autism and inattention measures and lower scores for adaptive functioning, compared to those in the TD-enriched cluster; Participants with ADHD and ASD in the ADHD-enriched clusters had significantly higher IQ and hyperactivity, respectively, compared those in the TD-enriched cluster. These results further highlight the importance of a dimensional approach to characterization of neurodevelopmental conditions.

Our analytical pipeline considered measures of morphology for brain regions in isolation, as done in previous literature, but also integrated information about statistical associations among pairs of regions. This was done by using participant-level measures of deviance from regional correlations. This approach was motivated by the wide-spread consensus that autism and ADHD are associated with pervasive differences across brain networks (33, 34) instead of differences in isolated regions of the brain. Our results showed significant differences between the data-driven clusters in these association measures in regions that have been previously associated with neurodevelopmental conditions including the fronto-temporal and cingulate regions (82). For cortical volume, our pattern of findings was consistent with one of the few previous studies examining the structural covariance of gray matter volume in ADHD (83). Increased structural associations were observed in the ADHD- compared to TD-enriched group in regions including the middle temporal and cingulate gyri, regions that comprise the default mode network (84) which is frequently implicated in ADHD (85). Differences in connections involving subcortical regions were also reported between the ADHD- and TD-enriched groups; structural alterations in subcortical regions are frequently reported in the ADHD literature, potentially underlying problems in domains such as emotional regulation (86). For cortical thickness, increases in structural connectivity in the TD- compared to ADHD-enriched group were observed in fronto-temporal connections, which has been shown to be related to inhibitory control in individuals with ADHD (87).

Our results should be interpreted in the context of significant cluster differences in age and sex ratios. In particular, the TD-enriched cluster was significantly older than those in the ADHD-enriched cluster, and contained fewer males. Given that the data were corrected for age prior to analyses, the age differences between clusters may be related to differences in neurodevelopmental trajectories [e.g., differences may decrease with age (2, 32, 37)], and specifically age-related differences in presentation of ADHD-like symptoms. Longitudinal studies are needed to future investigate this issue. Sex differences in clusters are not surprising as these have been previously reported in the prevalence and expression of ASD and ADHD (88–93). These differences may also be related to increased representation of male participants in the autism and ADHD groups.

Overall, our results contribute to the emerging body of literature motivating a shift away from broad diagnostic labels for autism and ADHD towards increased precision at phenotypic, cognitive, and biological levels. Data-driven approaches, when appropriately validated and replicated, can contribute to this precision by identifying trans-diagnostic biological and cognitive features that are targets for intervention and/or markers of treatment response. Our findings, for example, when replicated, can potentially identify children who, regardless of diagnosis, may benefit from interventions focused on ADHD symptoms such as difficulties with response inhibition.

## Limitations

The results of our study must be interpreted in the context of three limitations. First, our analyses focused on structural MRI data; future multi-modal analysis (e.g., including fMRI) may provide additional insights into the variability across neurodevelopmental conditions. Second, replication of the procedure on larger independent samples is also needed. Third, although the complexity of our analytic pipeline allowed for the integration of regional associations, future studies are needed to further clarify the interpretation of the findings in terms of specific neurobiological features involved.

## Data Availability

The data analyzed in this study is subject to the following licenses/restrictions: The data that support the findings of this study are available as per Ontario Brain Institute data access policy. Requests to access these datasets should be directed to https://pond-network.ca.
