# Supplementary material for: Investigating heterogeneity across autism, ADHD, and typical development using measures of cortical thickness, surface area, cortical/subcortical volume, and structural covariance
Source: Front Child Adolesc Psychiatry. 2023 Sep 27;2:1171337. doi: 10.3389/frcha.2023.1171337 (PMC11747914; doi:10.3389/frcha.2023.1171337)
Supplement: Supplementary file 1 [file Datasheet1.pdf]

SUPPLEMENTARY MATERIALS

DETAILS OF THE ANALYTICAL PIPELINE

The analytical pipeline used for clustering is comprised of the six steps below:

***1) Compute single participant structural association graphs*.** While structural covariance is typically computed for the sample as a whole, our pipeline computed statistical association matrices for each participant. To do this, we fit a linear regression model for each pair of brain regions across all participants. If the linear association between the two regions was significant (R^2>0.1, p<0.0001; zero similarity, otherwise), the Gaussian transformed value of the Cook's distance[1] was computed for each participant as the participant-level measure of structural covariance. Cook's distance integrates the residual and leverage and quantifies the dissimilarity of a participant from the rest of the sample with respect to a regression line[2]. The Gaussian transform was computed with a zero mean and a standard deviation equal to the 75th percentile of the data. Repeating this procedure for all region pairs and for each participant, we constructed a graph with regions as nodes and cook's distance in form of similarity as edge weights. This process was performed for area, cortical thickness, cortical volume, and subcortical volume independently, resulting in four statistical association graphs for each participant (4N, 76-node graphs).

2) Compute a participant similarity graph. The statistical association graphs from step 1 were compared using a Gaussian-transformed Lambda distance[3,4] (square root of the sum of the squares of the eigenvalues) to compute the similarity between pairs of participants for each brain measure. To do this, for each graph, eigenvalues were calculated using the Laplacian method, and the square root of the sum of the eigenvalues was used to obtain the Lambda distance. To transform the distance to similarity, a Gaussian transform with mean and standard deviation of the data was used. The result was a graph (per measure) representing the similarity of participants.

This step resulted in a single graph for each measure with participants as nodes and similarities as edge weights (4, N node graphs).

3) Merge similarity matrices. The similarity graphs for cortical thickness, surface area, cortical volume, and subcortical volume were fused using Similarity Network Fusion (SNF)[5] a graph-based data fusion method, resulting in a single, integrated graph with participants as nodes and similarities as edge weights. The optimization method proposed by Markello et al. was used to optimize the parameters μ and K, with the ranges $\mu \in [0.3, 0.8]$ and $K \in [5,45]$.

4) Integrate structural association similarity with regional brain measures. The similarity graph in step 2 was built using statistical associations between regions with respect to our brain measures. The objective of this step was to integrate this matrix with regional measures of cortical thickness, surface area, and cortical and subcortical volume. This was accomplished using a Graph Neural Network (GNN). To this end, each node (participant) of the participant similarity graph (step 3) was augmented with the cortical thickness, surface area, and cortical and subcortical data as features. A GNN model called the Adversarially Regularized Graph Autoencoder for Graph Embedding[6] was then used to obtain lower-dimensional representation for the data. This GNN model is a graph convolutional autoencoder with an additional cost function as an adversarial discriminator, enforcing a distribution match between the embedding and the original data[6]. This approach uses a graph convolution model to integrate the graph structure with the features and generates lower dimensional representation for the output. The parameters of the GNN were set based on the guidance provided by Shirui Pan et al[6] (32-neurons in the hidden layer, 16-neurons in the embedding layer, 16 and 64 neurons for the discriminators, 200 iterations for training with a learning rate of 0.001). The output of the GNN was a 16-dimensional vector for each participant. K-means clustering[7] was used to cluster the compact representation of each participant derived in step 3. To reduce sensitivity of the clustering solution to choice of parameters, this step was repeated with varying the number of clusters and SNF parameters, and consensus between

resultant clusters was used to drive final clusters[27]. Similar to the proposed work by Wang et al[5], the consensus with 95th percentile was used during grid-search of SNF to further eliminate any noise/random connectivity.

***5) Enhancing stability*.** To ensure stability of the clustering solution and to reduce the randomness introduced in GNN weight training, we repeated the entire analysis pipeline until the mean squared error between consecutive similarity matrices from step 4 was smaller than 0.00001 (steps of 500; 15,000 iterations).

***6) Final clustering*.** The final similarity matrix was constructed as the percentage of iterations where the participants shared the same cluster label in step 4 (probability of co-assignment). This similarity matrix was used as the final representation to perform hierarchical clustering. The Davies Bouldin score[8], Silhouette Coefficient[9], and Calinski-Harabasz score[10] were used to find the optimal number of clusters.

References

1. Cook, R. D. Detection of Influential Observation in Linear Regression. *Technometrics* **19**, 15–18 (1977).
2. Field, A. P. *Discovering statistics using IBM SPSS statistics*. (Sage Publications Inc, 2018).
3. Van Mieghem, P. *Graph Spectra for Complex Networks*. (Cambridge University Press, 2010). doi:10.1017/CBO9780511921681.
4. Sanfeliu, A. & Fu, K.-S. A distance measure between attributed relational graphs for pattern recognition. *IEEE Trans. Syst. Man Cybern.* **SMC-13**, 353–362 (1983).
5. Wang, B. *et al.* Similarity network fusion for aggregating data types on a genomic scale. *Nat. Methods* **11**, 333–337 (2014).
6. Pan, S. *et al.* Adversarially Regularized Graph Autoencoder for Graph Embedding. in *Proceedings of the Twenty-Seventh International Joint Conference on Artificial Intelligence* 2609–2615 (International Joint Conferences on Artificial Intelligence Organization, 2018). doi:10.24963/ijcai.2018/362.
7. Likas, A., Vlassis, N. & J. Verbeek, J. The global k-means clustering algorithm. *Pattern Recognit.* **36**, 451–461 (2003).
8. Davies, D. L. & Bouldin, D. W. A cluster separation measure. *IEEE Trans. Pattern Anal. Mach. Intell.* **1**, 224–227 (1979).
9. Rousseeuw, P. J. Silhouettes: A graphical aid to the interpretation and validation of cluster analysis. *J. Comput. Appl. Math.* **20**, 53–65 (1987).
10. Calinski, T. & Harabasz, J. A dendrite method for cluster analysis. *Commun. Stat. - Theory Methods* **3**, 1–27 (1974).

SUPPLEMENTARY TABLES

Supplementary Table 1. Scanner parameters for neuroimaging data. TR: Repetition Time, TE: Echo Time, FA: Flipped Angle, FOV: Field of View.

Location	Scanner	n	Sequence	TR (ms)	TE (ms)	FA (°)	FOV (mm)	Voxel size (mm)	Scan time (min)
Hospital for Sick Children	3T Tim Trio	280	MPRAGE	2300	2.96	9	192×240 ×256	1	5
Hospital for Sick Children	3T PrismaFIT	395	MPRAGE	1870	3.14	9	192×240 ×256	0.8	5
Queen's University	3T Tim Trio	113	MPRAGE	2300	3.14	9	192×240 ×256	0.8	6.2

Supplementary Table 2. List of regions included in the analysis.

**Cortical Area**	
Gyrus Rectus	Precuneus
Olfactory Cortex	Superior occipital gyrus
Superior frontal gyrus orbital part	Middle occipital gyrus
Superior frontal gyrus medial orbital	Inferior occipital gyrus
Middle frontal gyrus orbital part	Calcarine fissure and surrounding cortex
Inferior frontal gyrus orbital part	Cuneus
Superior frontal gyrus dorsolateral	Lingual gyrus
Middle frontal gyrus	Fusiform gyrus
Inferior frontal gyrus opercular part	Heschl gyrus
Inferior frontal gyrus triangular part	Superior temporal gyrus
Superior frontal gyrus medial	Middle temporal gyrus
Supplementary motor area	Inferior temporal gyrus
Paracentral lobule	Temporal pole superior temporal gyrus

Precentral gyrus	Temporal pole middle temporal gyrus
Rolandic operculum	Parahippocampal gyrus
Postcentral gyrus	Anterior cingulate and paracingulate gyri
Superior parietal gyrus	Median cingulate and paracingulate gyri
Supramarginal gyrus	Posterior cingulate gyrus
Angular gyrus	Insula
**Subcortical**	
nucleus accumbens / ventral striatum	CA1
precommissural putamen	subiculum
precommissural caudate	CA4DG
postcommissural caudate	CA2CA3
postcommissural putamen	X
lateral geniculate nucleus (LGN)	CM
medial geniculate nucleus (MGN)	Vermal III
anterior nuclei	Vermal IV
central nuclei	Vermal V
lateral dorsal	Vermal VI
lateral posterior	Vermal VIIA
medial dorsal	Vermal VIIB
pulvinar	Vermal VIIIA
ventral anterior nucleus	Vermal VIIIB
ventral lateral nucleus	Vermal IX
ventral posterior nucleus	Vermal X
Vermal I_II	stratum
III	Alv
IV	Fimb

V	Fornix
VI	Mam
Crus I	amygdala
Crus II	striatum
VIIB	gp
VIIIA	thalamus
VIIIB	Pituitary
IX	

Supplementary Table 3. Number of participants failing quality control by diagnosis.

	ASD	ADHD	TD
**Total (age 5-23)**	314	198	149
**Passed QC CIVET**	271	175	136
**Passed QC MAGeT**	262	171	132

Supplementary Table 4. Number of participants failing quality control by age.

	5-10 years(n=226)	Older than 10 years(n=512)
**Failed QC CIVET**	41 (18.14%)	38 (7.42%)
**Failed MAGeT**	17 (7.52%)	23 (4.49%)

Supplementary Table 5. Participant characterization on the NEPSY and stop-signal Task.

Measure	Autism (n=102)	ADHD (n=129)	TD (n=93)	Group effect (p-value)
**NEPSY - Affect Recognition**	8.50(6)	10(4)	11(4)	<0.0001 (TD>ADHD>ASD)
**NEPSY - Memory For Faces**	8(5)	9(4)	11(6)	<0.0001 (TD>ADHD>ASD)
**Stop signal - Response Inhibition**	267.3(136)	324.0(160.4)	251.8(104.2)	<0.0001 (ADHD>ASD>TD)
**Stop signal- Sustained Attention**	640.0(210.5)	645.1(167.1)	648.4(164.64)	0.878

Supplementary Table 6. Medication use in the sample.

Medication	ADHD	Autism	TD	ADHD-Enriched	TD-Enriched
**ABILIFY**	21	27	0	32	16
**ACCUTANE**	0	1	0	0	1
**ADDERALL**	7	0	0	5	2
**ALESSE**	9	10	1	14	6
**ANTIBIOTICS**	0	0	1	1	0
**ANXIETY**	0	1	0	1	0
**APO SERTRALINE**	0	1	0	1	0
**AQUEOUS**	0	0	1	1	0
**B12**	0	2	0	1	1
**BENADRYL**	0	1	0	1	0
**BIPHENTIN**	16	16	0	17	15
**BIRTH CONTROL**	0	1	1	1	1
**BUSCOPAN**	0	1	0	0	1
**CALCIUM**	0	1	0	1	0
**CALCIUM & VITAMIN D**	0	1	0	0	1
**CBD OIL**	0	1	0	0	1

**CITALOPRAM**	0	1	0	0	1
**CLARITIN**	0	1	0	1	0
**CLONIDINE**	1	5	0	3	2
**CLONAZEPAM**	0	1	0	1	0
**CORTISONE CREAM**	0	1	0	1	0
**DESMOPRESSIN**	0	2	0	0	2
**DEXEDRINE**	1	1	0	0	2
**EPI-PEN**	0	1	0	1	0
**FLOVENT**	6	7	0	7	6
**FLUOXETINE**	2	7	0	3	6
**GUANFACINE HCL**	0	1	0	1	0
**HYDROXYUREA AREA**	0	0	1	1	0
**HYDROXYCHLOROQUINE**	0	1	0	0	1
**INTUNIV**	3	8	0	2	9
**INVEGA**	0	1	0	0	1
**LACTULOSE**	0	1	0	0	1
**LAMICTAL**	0	2	0	0	2
**LATUDA**	0	1	0	0	1
**LAX-A-DAY**	0	1	0	1	0
**LUVOX**	0	2	0	1	1
**MARVELON**	0	1	0	0	1
**MELATONIN**	0	5	0	3	2
**METFORMIN**	0	1	0	1	0
**METHYLPHENIDATE**	1	11	0	7	5
**MOMETASONE FUROATE**	0	0	1	1	0
**MONTELUKAST**	0	0	1	1	0

**MULTIVITAMIN**	0	2	0	0	2
**OLANZAPINE**	0	1	0	0	1
**OMEGA 3**	0	1	0	1	0
**OMEGA FATTY ACID**	0	1	0	0	1
**OXYTOCIN**	0	1	0	0	1
**PAROXETINE**	0	1	0	1	0
**PMS-CETIRIZINE**	0	1	0	1	0
**PROPRANOLOL**	0	2	0	1	1
**QVAR**	0	1	0	0	1
**RAN-RISPERIDONE**	0	1	0	1	0
**REACTINE**	0	1	0	0	1
**RILUZOLE**	0	1	0	0	1
**RISPERDAL**	0	2	0	1	1
**RISPERIDONE**	2	10	0	3	9
**RITALIN**	2	5	0	4	3
**SALBUTAMOL INHALER**	0	1	0	1	0
**SEROQUEL**	0	2	0	0	2
**SERTRALINE**	0	2	0	0	2
**SONDAZ ESCITALOPRAM**	0	1	0	1	0
**STEROID FOR ASTHMA**	0	1	0	0	1
**STOOL SOFTENER**	0	1	0	0	1
**STRATTERA**	2	7	0	3	6
**SYMBICORT**	0	1	0	0	1
**TIDEGLUSIB**	0	1	0	0	1
**TIDEGLUSIB**	0	1	0	1	0
**TRAZODONE**	0	1	0	1	0

**TRYPTAN**	1	0	0	1	0
**TRYPTOPHAN**	0	1	0	0	1
**TYLENOL**	0	1	0	0	1
**VALACYCLOVIR**	0	1	0	1	0
**VENTOLIN INHALER**	0	1	0	0	1

SUPPLEMENTARY FIGURES

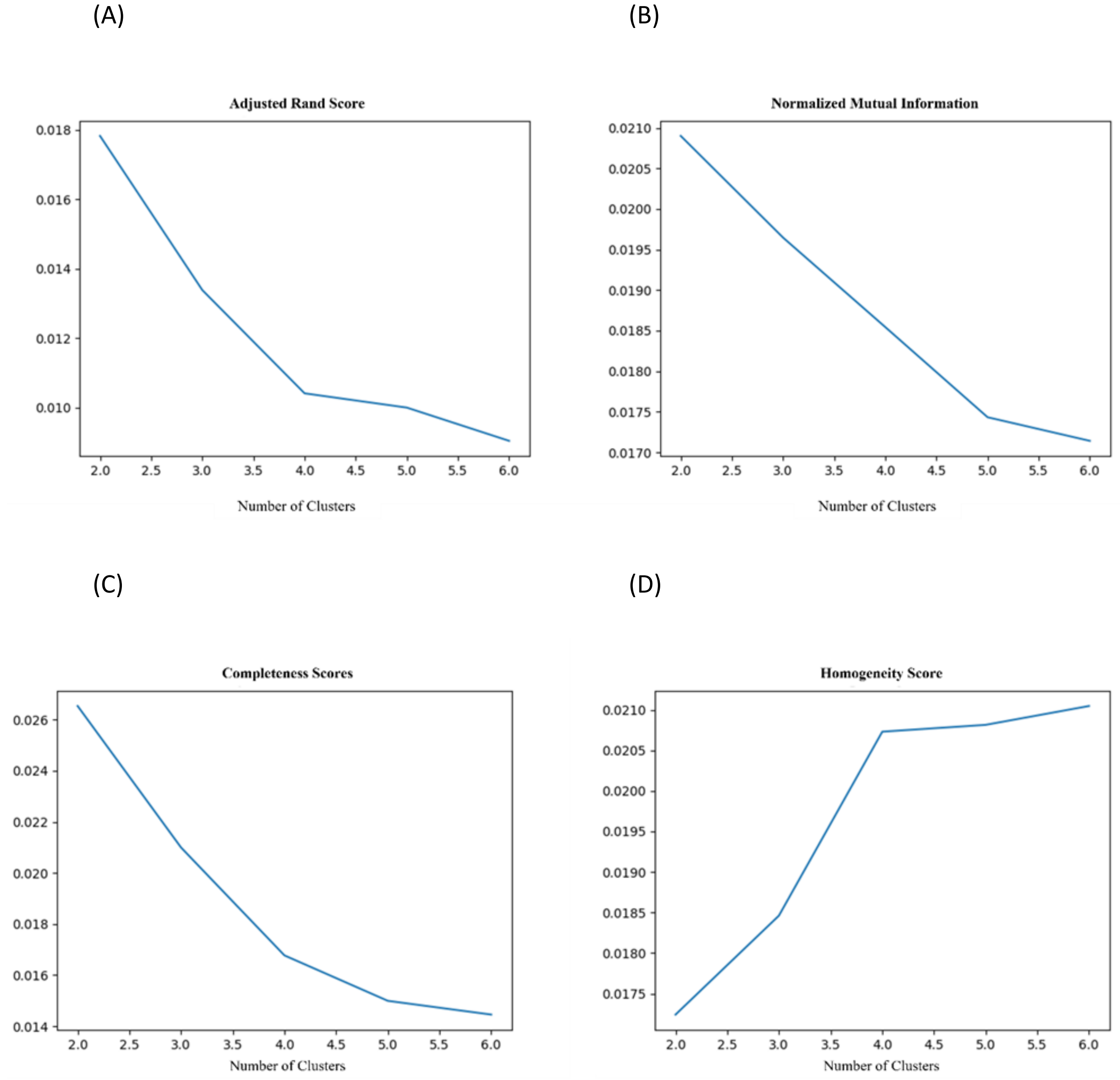

Supplementary Figure 1: Adjusted rand score (A), normalized mutual information (B), completeness score (C), and homogeneity score (D) quantifying the alignment between clusters and diagnostic groups.
